# Experiences of Ex-Smokers Serving as Peer Supporters in an Instant Messaging-Based Smoking Cessation Program: A Qualitative Study

**DOI:** 10.3390/nursrep16050170

**Published:** 2026-05-18

**Authors:** Ziqiu Guo, Tzu Tsun Luk, Xue Weng, Jung Jae Lee, Yongda Wu, Shengzhi Zhao, Derek Yee Tak Cheung, Agnes Yuen Kwan Lai, Henry Sau Chai Tong, Vienna Wai Yin Lai, Tai Hing Lam, Man Ping Wang

**Affiliations:** 1 School of Nursing, The University of Hong Kong, Hong Kong SAR, China; 2 Institute of Advanced Studies in Humanities and Social Sciences, Beijing Normal University at Zhuhai, Zhuhai 519087, China; 3 Children’s Hospital of Eastern Ontario Research Institute, Ottawa, ON K1H 8L1, Canada; 4 School of Public Health, The University of Hong Kong, Hong Kong SAR, China; 5 School of Nursing and Health Studies, Hong Kong Metropolitan University, Hong Kong SAR, China; 6 Hong Kong Council on Smoking and Health, Hong Kong SAR, China

**Keywords:** ex-smoker, experience, peer support, smoking cessation, mobile health, qualitative study

## Abstract

**Objectives:** To explore the experiences of ex-smokers acting as peer supporters delivering chat-based instant messaging smoking cessation support in a post- randomized controlled trial qualitative study. **Methods**: We used purposive sampling to recruit 21 ex-smokers, who had previously provided chat-based instant messaging behavioral support to smokers in Hong Kong. Individual online semi-structured interviews were conducted. All interviews were audiotaped and transcribed verbatim. The transcripts were analyzed using inductive thematic analysis following Braun and Clarke’s six-phase framework. **Results:** Five themes were identified: stepping into the supporter role as ex-smokers, contributions beyond facilitating smoking cessation, mutual benefits as ex-smoking peer supporters, challenges in providing support, and the potential of mobile health-based peer support. Participants identified experiential sharing as a key mechanism for engaging motivated smokers, although its impact was limited by low user responsiveness. They described extending their support beyond smoking cessation (e.g., stress relief and healthier lifestyles), and reported mutual benefits, such as enhanced happiness, a positive attitude, and prevention of smoking relapse. Nevertheless, participants’ ability to effectively support smoking was challenged by limited interaction in the mHealth setting, difficulties offering timely responses, and uncertainty about how to handle complex personal situations. They suggested that future mobile health peer support can consider involving famous people as peer supporters, using personalized messaging, and adding incentives in the program to increase smokers’ motivation. **Conclusions:** Ex-smoking peer support in mHealth settings appears feasible and mutually beneficial, but its effectiveness may be constrained by low engagement and unclear role expectations.

## 1. Introduction

Peer support is the provision of emotional, instrumental, and informational support by people who share similar experiences with the recipients. Ex-smokers as peer supporters could provide better emotional and informational support for smoking cessation (SC) than current or non-smoking peer supporters, but they have been rarely included in SC services and interventions [1]. A 2023 meta-analysis of five trials (four randomized controlled trials [RCT] or one pilot RCT) has shown that SC intervention led by ex-smoking peer supporters was effective in increasing the biochemically smoking abstinence at 3 to 9 months follow-up [1]. The effectiveness of ex-smoking peer support for SC might be affected by the peers’ performance, which may depend on their support skills, personal experiences, and motivation to assist others [2].

Understanding the experiences of ex-smoking peer support is important for increasing the effect size of such interventions. Our literature review found one related study exploring the ex-smoking peer supporters’ experiences in supporting the smokers with psychiatric illness after a professionally led intervention [3]. This study has shown that peer roles are multifaceted and extend beyond merely providing support for SC. These challenges might arise from the dual responsibility of supporting both SC and psychiatric illness, and the inherent difficulty in defining appropriate boundaries with participants. Notably, the experiences reported in the study might differ from those of peer supporters who take a leading role in providing SC support, considering ex-smoking peer supporters here were included in the professionally led intervention, which may have influenced the support experience. Additionally, these experiences were ex-smoking peer supporters who provided group-based, face-to-face support.

Mobile health (mHealth) offers new opportunities to engage peer supporters in providing support, enabling them to offer assistance on flexible schedules without the need for face-to-face meetings and travel. Delivering support through mHealth might differ from traditional face-to-face approaches. A Cochrane review has shown the experience of using mHealth to deliver services by health professionals. It has identified the specific challenges associated with mHealth-based support, including skepticism from support recipients, insufficient contact with support recipients, particularly when recipients do not actively initiate the conversation, and ambivalent supporting hours [4]. Although mHealth-based peer support is increasingly common for SC [5,6], existing qualitative evidence on ex-smoking peer supporters’ supporting experience is scarce, particularly in mHealth contexts where they play a primary support role.

In Hong Kong, the smoking prevalence is the lowest in the world (9.1% at 2023), but the proportion of smokers with no previous quit attempt nor willing to quit is high (61.5%) [7]. To promote SC, the government has launched several free, nurse-led SC services. The Department of Health operated territory-wide telephone quitlines or clinics that provide SC counseling and pharmacotherapy in the community. The Hospital Authority also integrates SC services into routine outpatient care. Although these services are accessible and free, only a small proportion of smokers use the services. mHealth modalities have been shown to be an effective means of delivering continuous and personalized SC support to expand cessation services [8]. Compared with professionals who provided mHealth-based SC support, smokers expressed a willingness to accept support from ex-smokers, as they might be more empathetic [9]. We have developed a program to proactively reach smokers with low motivation to quit and evaluated the effect of mHealth-based support from ex-smoking peer supporters on smoking abstinence [6]. A small but non-significant increase in smoking abstinence was observed in smokers receiving mobile-based ex-smoking peer support (RR 1.32, 95% CI 0.88, 1.99). Smokers rated the effectiveness of mobile-based ex-smoking peer support in promoting quitting as moderate (mean score 6.20 out of 10). Considering that the support effect might be associated with supporters’ performance, this study aimed to explore the experiences of ex-smokers serving as peer supporters providing SC support via instant messaging.

## 2. Materials and Methods

### 2.1. Study Design and Ethical Considerations

This was an exploratory qualitative study conducted after completion of a trial in which ex-smokers served as peer supporters for SC via mHealth. The protocol was approved by the Institutional Review Board of the University of Hong Kong/Hospital Authority Hong Kong West Cluster (UW 21-288). Online written consents were obtained after explaining the study. The study reporting followed the Consolidated Criteria for Reporting Qualitative Studies [10].

### 2.2. Participants

We purposefully recruited ex-smoking peer supporters who had previously provided mHealth-based behavioral support in our trial to attend online interviews. The eligibility criteria for being ex-smoking peer supporters in the trial were aged 18 years or above, having quit smoking for ≥1 year, being willing to provide SC support via instant messaging apps, and having completed the required training by the team. Ex-smoking peer supporters were briefly trained (around 90 min) before they joined the trial. The training included (1) a brief introduction of the program, (2) brief information on smoking hazards of traditional and alternative tobacco products, (3) the role and rules of ex-smoking peer supporters, and (4) a brief introduction of positive psychological exercise and counseling skills based on positive psychology. Ex-smoking peer supporters supported smokers by sharing quitting experiences and providing encouragement within 3-person instant messaging groups (i.e., one ex-smoking peer supporter, one smoker, and one SC advisor) in the trial. The ex-smoking peer supporters first introduced themselves to arouse smokers’ interests upon joining the 3-person chat groups. When smokers asked questions, ex-smoking peer supporters messaged their quitting experiences and encouragement. The duration of support lasted for 3 months for one smoker. Each ex-smoking peer supporter ultimately joined an average of 21 groups to support smokers. A small honorarium of HK$100 (US$12.8) was provided for ex-smoking peer supporters after supporting one smoker for 3 months. Ex-smoking peers had the right to withdraw from the study at any time without providing a reason or penalty (but informing the team). In March to May 2022, a total of 21 ex-smoking peer supporters completed the interviews. Sampling continued until data saturation was reached, with no new themes emerging in the final interviews.

### 2.3. Data Collection

Individual semi-structured interviews with open-ended questions were conducted in Cantonese through online Zoom meetings (https://www.zoom.com/en/products/virtual-meetings/ [access on March to May, 2022])by two researchers (ZG and QS), both female SC researchers trained in qualitative methods. Zoom was chosen because participants were familiar with online communication through brief training, and to enable data collection in the COVID-19 pandemic context. Each interview lasted for around 40 min, which was sufficient to complete the interview in depth without over-burdening participants. The interviews were audio or video-recorded (depending on participants’ consent) with participants’ permission. Reflective field notes (i.e., mainly initial impressions) were written by an interviewer (ZG) to facilitate the data analysis process [11]. The qualitative interview guides covered participants’ experiences and suggestions for the potential refinement of the intervention. Probes such as “Tell me more” “Can you give us an example?” were used to elicit more detailed responses. The detailed interview guideline is in Appendix A.

The interviewers had no prior personal relationship with participants. We recognized that participants might tend to emphasize positive experiences, as the interviewers were from the research team that had previously invited them to provide support. Therefore, at the start of each interview, the interviewers explained the aims of the interviews and emphasized that there were no right or wrong answers for the questions, and the participation or responses would not affect the current or future involvement in the program.

### 2.4. Data Analysis

All audio/video recordings were transcribed verbatim and analyzed in the original Cantonese. An inductive thematic analysis, as described by Braun and Clarke, was conducted to identify patterns in participants’ experiences [12]. The analysis was primarily at a semantic level, focusing on the explicit content of participants’ accounts. First, all transcripts were read line-by-line to generate preliminary thoughts on the data. Second, passages related to the questions were coded. The codes were generated from data instead of a pre-existing coding framework and focused primarily on participants’ explicit meaning. Third, codes that shared similar meanings were collated as initial themes. Fourth, these initial themes were reviewed against the coded data and the full dataset. Two researchers closely discussed all differences in the coding decision by re-analyzing the transcript’s extracts. Fifth, after refining, the final themes were defined and named. Finally, illustrative quotations were selected for reporting. Data was managed and coded using NVivo 12 (QSR International Pty Ltd., Melbourne, Australia) to organize transcripts, codes, and themes. All the analyses were conducted in Cantonese, and selected quotes were translated into English for reporting.

### 2.5. Rigor

Several strategies, aligned with established qualitative quality criteria (credibility, dependability, confirmability, and transferability) [13], were used to ensure the rigor of the analysis. To enhance credibility and dependability, a researcher cross-checked transcripts against the audio/video recordings to ensure transcription accuracy. The selected quotes were forward-backward translated by bilingual researchers who were proficient in Cantonese and English to further support the credibility and dependability in a bilingual context. To enhance the confirmability, external validation was sought through peer debriefing with experienced qualitative researchers (JJL andXW), who provided critical feedback that helped to refine the coding and thematic structure. To enhance the transferability, we provided a detailed description of the study context, characteristics of the investigators, participants, sampling strategies, data collection, and data analysis procedures. Member checking with participants was not conducted because of the budget constraints. Instead, all the above strategies were used to enhance the overall trustworthiness of the analysis.

## 3. Results

Table 1 shows that of 21 participants, 16 were male, 11 attained tertiary or above education, 6 held a monthly income of less than HK$10,000 (US$1 = HK$7.8), and 16 had full-time jobs.

Five themes were identified from the analysis: (1) Stepping into the supporter role as ex-smokers, (2) Contributions beyond facilitating SC, (3) Mutual benefits as ex-smoking peer supporters, (4) Challenges in providing support, and (5) Potential of mHealth-based peer support. Summaries of themes and sub-themes are shown in Table 2.

### 3.1. Theme 1: Stepping into the Supporter Role as Ex-Smokers

#### 3.1.1. Establishing Legitimacy Through Experience

Participants described transitioning into the supporter role as a process of legitimizing their experiential knowledge and adapting it to a semi-structured intervention context. Through introducing themselves while joining the group, sharing past experiences to support smokers, encouraging smokers, and echoing the SC advisor’s regular messages when smokers had no questions/responses, they sought to transform personal experiences of smoking/cessation into credible resources for others.

*“I felt my support was helpful for those who had ever responded to me because some smokers shared similar experiences with me. Like me, most smokers cannot successfully quit at one try. I talked to them and shared with them how I finally made it. I felt they understood me and would have a try.”*

(Case 20, male, aged 23)

The quotation has shown that young supporters reframed his multiple failed quit attempts as a resource. By emphasizing that he finally made it, the participant positioned himself as a legitimate role model whose success is attainable.

#### 3.1.2. Adapting to a Semi-Structured Intervention Context

Given the low engagement from smokers in an instant messaging-based intervention, participants also developed several informal techniques to engage smokers. These included asking about smokers’ quitting progress to facilitate interaction, expressing empathy, using down-to-earth messaging, and linking smokers’ quitting progress to real-life events (e.g., the weather, the 2020 Summer Olympics). These indicated that participants actively adapted themselves to fit into the program structure.

Participants felt that the effectiveness of their support was related to smokers’ responsiveness. Most of them reported that only about 20–30% of smokers interacted with them, and that most smokers never responded. Consequently, they perceived their overall impact on SC was positive but limited.

*“To be honest, most participants had never responded in the group when I shared my quitting experiences. A few of the participants only replied with thanks and emojis. According to participants’ responses status, I felt my support was not very helpful”*

(Case 21, male, aged 36)

This reflected the early tension in the peer role. Ex-smoking peer supporters perceived themselves as helpful when engagement occurred, but less helpful when no responsiveness occurred.

### 3.2. Theme 2: Contributions Beyond Facilitating SC

After settling into the supporter role, most participants perceived their contribution more than SC alone. More than acting as SC advisors, participants described that they helped smokers alleviate stress and further promote a healthy lifestyle, which expanded their remit to a wider wellbeing of smokers.

*“One smoker said she was stressed because of losing her job in the COVID-19 pandemic. Although I did not have rich life experience, I comforted her and helped her to reduce the stress.”*

(Case 17, female, aged 28)

*“I encouraged smokers to build a daily activity. Some smokers enjoyed smoking while watching TV and lying on the couch. I encouraged them to walk in the garden where smoking is forbidden. Walking in the garden could also help them to breathe fresh air. I believed this could help them activate their bodies and build a healthy lifestyle.”*

(Case 7, male, aged 50)

All these reflected that ex-smokers reframed their role from narrowly assisting with SC to fostering overall well-being. Meanwhile, the expansion of the role has set the stage for the mutual benefits they later reported (i.e., Theme 3).

### 3.3. Theme 3: Mutual Benefits as Ex-Smoking Peer Supporters

#### 3.3.1. Prevented Smoking Relapse

Participants commented that supporting the smokers prompted them to continually discuss the benefits of quitting. This reminded them of their original reasons for quitting and reinforced their determination to avoid relapse.

*“Supporting smokers to quit smoking kept on reminding me of the reasons that I wanted to quit. These helped me to keep on staying abstinent.”*

(Case 13, female, aged 31)

While supporting smokers through the quitting process, participants recognized that relapses could easily occur at any time and that their current non-smoking status could revert to smoking. Another participant described a similar awareness that sharing his quitting history linked to relapse prevention.

*“Sometimes sharing my experiences with smokers reminded me there was a chance of relapse, and I should not smoke back.” *

(Case 6, male, aged 29)

The specific mechanism of relapse prevention may have been that giving advice continually reactivated their own motivation to quit. Another possible mechanism was that supporting smokers to quit required participants to act as role models who maintained abstinence, thereby consolidating their non-smoker identity.

#### 3.3.2. Enhanced Happiness

Beyond relapse prevention, participants expressed happiness in being able to help smokers, particularly when they received positive feedback. A younger supporter particularly described this benefit:

*“I was happy of being an ex-smoking peer supporter. I was in Hong Kong alone and just graduated for a short period. I never thought I could help people.”*

(Case 20, male, aged 23)

This reflected that being able to help others supported a more valued self-image.

#### 3.3.3. Formed a Positive Attitude

Some participants expressed that they developed a positive attitude because they believed it was an important trait for a supporter.

*“As an ex-smoking peer supporter, I must keep positive to support smokers. Because I believed a positive attitude would make them more likely to accept my advice.”*

(Case 8, male, aged 36)

In this case, participants perceived positivity is not only a personal trait but a strategy to ensure smokers are more willing to accept support. At the same time, the requirement to keep positive reflects an internalized expectation that supporters should manage their own emotions for the benefit of others.

### 3.4. Theme 4: Challenges in Providing Support

Despite the perceived benefits, participants identified several challenges that limited their ability to provide effective support and undermined their enthusiasm. These challenges are related to low interaction in the mHealth setting, difficulties in providing timely responses, and uncertainty in managing complex or sensitive situations.

#### 3.4.1. Challenges in Supporting Smokers with Low Interaction in the mHealth Setting

Low engagement from smokers was the most commonly reported challenge. Participants commented that smokers with low interaction might be less motivated to quit. They were concerned that an overload of SC-related information might make smokers feel pressured and cause reactance. Some participants also expressed unwillingness to force smokers to quit when they found smokers did not interact with them, since they perceived quitting smoking as a personal choice.

*“I have smoked and made several quit attempts. I knew the smokers’ feelings. Sometimes our behavior that pushes them to quit would have reversed effect. They might smoke if we always persuaded them to quit smoking. Therefore, I did not dare ask them if they had no responses in the 3-person chat groups.”*

(Case 15, female, aged 41)

Persistent silence in the chat groups also had an emotional impact on participants:

*“When I found no responses from the chat groups, I became less willing to message the chat groups either.”*

(Case 19, male, aged 50)

The low interaction from smokers not only reduces opportunities to help but also leads to some participants feeling frustrated and helpless, and ultimately reduces their passion for supporting smokers.

Some participants expressed that the mHealth setting caused difficulty in building a sense of intimacy with smokers. They perceived that the superficial interactions in the mHealth setting constrained their ability to fully realize the peer role they imagined.

*“Compared to face-to-face counseling, messaging in the chat group lacked intimacy and did not foster strong bonds.”*

(Case 2, male, aged 47)

Uncertainty about boundaries further complicated matters. Some participants worried that being too proactive or sharing certain information might have had a negative impact on the program. As a result, they tended to limit their contributions or only share briefly, often timing their messages to follow those sent by SC advisors to avoid overstepping perceived boundaries.

*“I thought I was one member of your (team). I was afraid to do much to cross the line or to affect the whole program. I asked the SC advisor to check if I could share some kinds of information in the chat group.”*

(Case 4, female, aged 60)

The quotation showed how participant negotiated their role between peer supporter and team member. They often tended to be more cautious. This might constrain the spontaneity and authenticity of peer support.

#### 3.4.2. Difficult to Respond in a Timely Manner

Participants noted that they could not reply to smokers in a timely manner due to their busy working schedules. Some participants expressed that their workload suddenly increased because of the outbreak of the 5th wave of COVID-19. Despite employing multiple strategies to ensure prompt responses, participants were concerned that smokers might not be able to maintain their efforts to quit smoking due to delayed replies.

*“Since I had my job, sometimes I could not read the messages in a timely manner. It was already hours later when I read the messages.”*

(Case 21, male, aged 36)

Participants worried that smokers’ motivation might fade if support was not provided when needed. This challenge highlights a structural mismatch between the ideal of “on-demand” support in mHealth and the realities of volunteer supporters with competing responsibilities.

#### 3.4.3. Difficult to Support Smokers Who Were in Specific Situations

Some participants commented that they did not know how to support smokers who were facing severe stress or complex life events.

*“I met a smoker who smoked because of the huge stress due to her family member having passed away. I did not know how to support her when she shared that with me.”*

(Case 17, female, aged 28)

In this situation, the supporters recognized that the smoker was using smoking as a stress relief, which exceeded her support ability. This reflected the limits of experiential knowledge and needed clearer guidance to referral pathways for complex psychosocial issues.

### 3.5. Theme 5: Potential of mHealth-Based Peer Support

Reflecting on their experience, participants proposed several refinements to strengthen mHealth-based peer support. Their suggestions, such as involvement of famous people, personalizing messaging, and offering incentives, can be understood as attempts to enhance intervention engagement.

#### 3.5.1. Famous People as Peer Supporters

Some participants suggested including famous people as supporters to increase smokers’ engagement in the program.

*“Smokers might be more willing to messaging with famous people…I remembered when I joined Quit to Win contest, Sinn Lap Man (local musician, singer, and actor) joined as a smoke-free ambassador. I felt he encouraged many people to join and attempt to quit smoking…However, they (those famous people) might not have time to join chat groups.”*

(Case 7, male, aged 50)

Participants acknowledged the practical constraints. Nevertheless, they believed that famous people are more appealing and might complement peer support, especially in recruitment and initial engagement.

#### 3.5.2. Personalized Messaging

Participants commented that personalized messaging might convey to smokers that their inquiries or concerns were being taken seriously.

*“Incorporating smokers’ surnames in the messaging would make them feel that their specific questions or concerns were being addressed. Text messages without surnames seem like announcements intended for a broad audience. Therefore, smokers felt less responsibility to reply to the announcements.”*

(Case 7, male, aged 50)

Personalization here is a signal that “this message is for you”. This strategy could potentially increase smokers’ engagement in chat groups and enhance the overall supportive effect.

#### 3.5.3. Increase Quit Motivation

Participants suggested that awards like supermarket coupons, cash, and public appraisal could be added to the program to increase smokers’ quitting motivation.

*“We might put those successful quitters’ names on the website of Quit to Win contest with their approval to praise them. Smokers might feel happy and encouraged.”*

(Case 7, male, aged 50)

*“I felt Hong Kong people like awards. Small awards like HK$50 supermarket coupon would let them treat quitting smoking seriously.”*

(Case 20, male, aged 23)

Participants perceived that incentives could reinforce smokers’ efforts and make quitting progress more tangible, which might ultimately contribute to quitting.

Overall, the five themes described the experiences of ex-smokers acting as peer supporters in an mHealth context. They legitimized their experiential knowledge and constructed a helper identity with a semi-structured intervention (Theme 1), extended their contribution to addressing smokers’ wellbeing (Theme 2), and gained mutual benefits (Theme 3). Meanwhile, they faced challenges (Theme 4), which undermined their perceived intervention effectiveness. Their suggestions for enhancing future interventions (Theme 5) can be seen as attempting to improve engagement within mHealth-based peer support interventions.

## 4. Discussion

This post-trial qualitative study is the first to explore ex-smokers’ experiences as peer supporters providing mHealth chat-based SC support. The experiences described by ex-smoking peer supporters were consistent with the requirements outlined in the brief training. Beyond these basic expectations, they often initiated additional interactions with smokers and shared real-time events to support quitting. All ex-smokers volunteered to join the trial for a very small incentive (US$12.80 for supporting one smoker over 3 months), and none withdrew from the study, indicating a strong commitment to helping smokers quit. Ex-smoking peer supporters perceived that their support not only helped smokers quit but also contributed to stress relief and the adoption of healthier lifestyles. The benefits reported are particularly important, as volunteer satisfaction is closely related to seeing positive results from their efforts [14]. These findings have shown that ex-smokers can feasibly be engaged as peer supporters via mHealth. However, our findings also showed a potential tension that is less emphasized in the existing literature. Ex-smokers experienced the pressure while they were smokers, so they tended to be cautious about pushing unresponsive or ambivalent smokers too hard. Experiential knowledge made ex-smokers credible but also caused ambivalence and constrained how they proactively intervene with smokers. Therefore, carefully integrating ex-smokers into SC services may reduce nurses’ workload and extend the reach and effectiveness of nurse-led SC support, but requires structural support and clear boundaries in the mHealth setting.

Ex-smoking peer supporters reported mutual benefits from taking on this role, which was consistent with the helper therapy principle [15]. By reflecting on their own challenging quitting experiences, peers were able to transform these experiences into valuable knowledge and support for others. This process was associated with enhanced self-esteem and improved mental well-being [16,17]. Notably, we further found that relapse prevention was a reciprocal benefit. Similar findings have been reported in the context of mental health support, where the act of helping others contributed to peer supporters’ own recovery [18]. The process of supporting others repeatedly articulated their reasons for quitting and described themselves as role models for smokers, which might consolidate ex-smokers’ own non-smoking identity and reinforce the ex-smoking peer supporters’ commitment to abstinence. Considering most smokers who achieve short-term abstinence easily return to smoking (relapse) [19,20], harnessing this reciprocal benefit might be a promising way for relapse prevention. It is worthwhile to explore whether integrating peer support roles into relapse prevention interventions can enhance the effectiveness of these programs and help ex-smoking peer supporters maintain a non-smoking identity. Future research should examine how these reciprocal mechanisms can be optimized in intervention design, potentially creating a mutually reinforcing cycle of support and sustained abstinence. It is worth noting that the implicit expectation of role models for smokers might also place pressure on ex-smoking peer supporters, who might also need ongoing support.

Despite these contributions and benefits, ex-smoking peer supporters described several challenges, including supporting smokers with low interaction via mHealth, difficulty responding to smokers in a timely manner while managing their own jobs, and handling specific situations. These challenges helped to explain the moderate effect of the support observed in the main trial [6], although this relationship cannot be directly established from the present qualitative data. The challenge of providing mHealth-based support to smokers who interact infrequently is consistent with findings from professionally led mHealth interventions, which similarly rely on recipients taking responsibility for their health and actively engaging with the support provided [4]. Our analysis found that non-responsive smokers were those who had no previous quit attempts or were less ready to quit [6]. A similar finding was observed that peer supporters were less helpful for smokers who are not ready to quit [21]. A lack of rapport and uncertainty about role boundaries in the mHealth context, as reported by ex-smoking peer supporters, may have further exacerbated the difficulty of engaging these smokers [22,23]. Future research should explore whether additional training, clear guidelines on role boundaries with structured supervision to reduce role ambiguity, and communication strategies for managing non-responsive /low-readiness smokers and building rapport, can help peer supporters feel more confident and effective in their role.

Ex-smoking peers perceived that the support should be personalized to enhance effectiveness. A previous RCT also showed that the personalized text messages doubled the biochemically sustained abstinence rate compared to those who received a non-personalized text message [24]. Personalization concept of mHealth for behavioral changes included user, system functionalities, information, and app properties [25]. Studies on identifying how and what specific characteristics or aspects are personalized to increase the effect size are needed. Consistent with previous studies that showed timely support effectively increased smoking abstinence [26], our ex-smoking peers also highlighted that the lack of timely support might undermine the support effect. With the continued advancement in artificial intelligence, chatbots that can provide timely support could also be developed as a supplementary tool for testing alongside peer support.

Finally, the specific context of this study should be considered when interpreting these findings. Smokers in Hong Kong are less ready to quit, which may partly explain the frequent non-responsiveness described by peer supporters. Moreover, each chat group includes both ex-smoking peer supporters and an SC advisor. This structure offered professional backup and might cause ex-smoking peers’ concern that they crossed the line. The fully remote support via mHealth modality might also lead to difficulty in building intimacy between peer supporters and smokers. Therefore, the experience of providing mHealth-based peer support may differ in other cultural and service contexts. These also need to be considered when adapting or scaling up similar programs.

### Limitations

The present study had several limitations. First, the relatively small sample might limit the information obtained. However, it is important to note that no emerging information was identified in the data before the end of the interviews, suggesting data saturation was reached. Second, the ex-smoking peers were recruited from those who had attended the trial. They successfully completed the peer supporters’ role with a small honorarium, which suggested that they might have been more motivated or committed to acting in the role of supporters. Therefore, our findings might not fully capture the perspectives of those less engaged ex-smoking peer supporters. Third, the ex-smoking peer supporters’ experiences might have been influenced by the interactions with SC advisors, who were also members of the 3-person chat groups involved in the trial. The presence of SC advisors might also have shaped how ex-smoking peer supporters understood and enacted their role. Experiences of ex-smoking peer support may therefore differ in interventions where peers operate without direct professional presence or within different services. Therefore, the perspectives might not fully represent the experiences of other supporters involved in different SC studies, and the generalizability to other supporters is uncertain. Fourth, interviews were conducted by researchers of the trial team, which may have introduced research and social desirability bias, with participants potentially emphasizing positive aspects of the program or under-reporting difficulties. We have clearly explained that there were no right or wrong answers before the interview started. Fifth, the ex-smoking peer supporters did not verify the final themes (member checking) due to budget constraints. This might affect the credibility of the results. Sixth, all the analyses were conducted in Cantonese, but the findings are reported in English using translated quotations. Some nuances, emotions, and culturally specific expressions might have been lost in translation. Finally, this qualitative study did not systematically report smoking reduction or abstinence outcomes of smokers who received support, which may limit the understanding of the direct link between participants’ experiences and the effectiveness of their support.

## 5. Conclusions

This study is the first to examine ex-smoking peer supporters’ experiences of delivering SC support via mHealth. Our findings highlight that while ex-smoking peer support in mHealth settings is perceived as meaningful and mutually beneficial, its effectiveness is shaped by engagement dynamics, role clarity, and contextual constraints. Future interventions should focus on enhancing smoker engagement, clarifying peer supporter roles, and integrating structured support mechanisms to maximize effectiveness.

## Figures and Tables

**Table 1:** Sociodemographic characteristics of ex-smoking peers who attended the interviews (n = 21).

Case	Sex	Age (Years)	Education	Monthly Income * (HK$)	Employment	Smoking Duration Before Abstinence (Years)	Tobacco Products Used Before Abstinence ^$^	Daily Cigarette Consumption Before Abstinence	Abstinence Duration (Years)
1	Male	41	Secondary	>50,000	Full-time job	20	Traditional cigarette + HTP + EC	3.5	2
2	Male	47	Secondary	<10,000	Retired	30	Traditional cigarette	30	2
3	Male	69	Tertiary or above	<10,000	Retired	40	Traditional cigarette	20	3
4	Female	60	Secondary	<10,000	Part-time job	2	Traditional cigarette	10	2.5
5	Male	30	Tertiary or above	20,000–30,000	Full-time job	8	Traditional cigarette	10	1
6	Male	29	Tertiary or above	20,000–30,000	Full-time job	7	Traditional cigarette + HTP	5	1
7	Male	50	Tertiary or above	>50,000	Full-time job	21	Traditional cigarette	15	8
8	Male	36	Tertiary or above	20,000–30,000	Full-time job	20	Traditional cigarette + HTP	10	1
9	Male	26	Secondary	30,000–40,000	Full-time job	7	Traditional cigarette + EC	20	2.5
10	Male	42	Tertiary or above	>50,000	Full-time job	25	Traditional cigarette	20	3
11	Male	64	Secondary	<10,000	Retired	20	Traditional cigarette	20	1.5
12	Male	49	Secondary	>50,000	Full-time job	20	Traditional cigarette	3.5	2
13	Female	31	Tertiary or above	30,000–40,000	Full-time job	4	Traditional cigarette	5.5	1.5
14	Male	47	Secondary	30,000–40,000	Full-time job	20	Traditional cigarette	24	4
15	Female	41	Secondary	<10,000	unemployed	28	Traditional cigarette	20	1.5
16	Female	33	Tertiary or above	20,000–30,000	Full-time job	10	Traditional cigarette	10	1.5
17	Female	28	Tertiary or above	30,000–40,000	Full-time job	2	Traditional cigarette	5	2
18	Male	68	Secondary	<10,000	Retired	50	Traditional cigarette	70	3
19	Male	50	Tertiary or above	20,000–30,000	Full-time job	20	Traditional cigarette	20	4.5
20	Male	23	Secondary	10,000–20,000	Full-time job	5.5	Traditional cigarette	10	1.5
21	Male	36	Tertiary or above	30,000–40,000	Full-time job	15	Traditional cigarette + EC	6.5	2

* US$1 = HK$ 7.8. ^$^ HTP: heated tobacco product; EC: electronic cigarette.

**Table 2:** Themes and sub-themes identified from thematic analysis of ex-smoking peers.

Themes	Sub-Themes
Stepping into the supporter role as ex-smokers	Establishing legitimacy through experience
	Adapting to a semi-structured intervention context
Contributions beyond facilitating smoking cessation	
Mutual benefits as ex-smoking peer supporters	Prevented smoking relapse
	Enhanced happiness
	Formed a positive attitude
Challenges in providing support	Challenges in supporting smokers with low interaction in the mHealth setting
	Difficult to respond in a timely manner
	Difficult to support smokers who were in specific situations
Potential of mHealth-based peer support	Famous people as peer supporters
	Personalized messaging
	Increase quit motivation

## Data Availability

The data in this study are available from the corresponding author on reasonable request due to privacy and ethical reasons.

## Public Involvement Statement

No public involvement in any aspect of this research.

## Guidelines and Standards Statement

The study reporting followed the Consolidated Criteria for Reporting Qualitative Studies.

## Abbreviations

SC	Smoking Cessation
RCT	Randomized Controlled Trial
mHealth	Mobile Health

## Appendix A. Qualitative Interview Guidelines

1. Ice-break questions: How did you quit smoking in the past?
2. Why did you join our program and become an ex-smoking peer supporter?
3. Could you describe your experience in the 3-person chat groups?
  - What did you do to help them quit smoking?
  - How did smokers respond to your supporting?
  - How would you describe the interaction between you and the smokers?
  - How do you view the role of an ex-smoking peer supporter?
4. How would you describe your supporting effect?
  - Apart from quitting smoking, in what other ways do you think your supported smokers?
  - What additional support do you think smokers still need?
5. In the process of supporting participants to quit smoking, what challenges did you face?
  - How did you overcome these challenges?
  - What support would help you overcome these challenges?
6. Could you please describe any positive personal impact that being a smoking cessation peer supporter has had on you?
7. What aspects of the program do you think could be improved?
